# Microbially influenced corrosion communities associated with fuel-grade ethanol environments

**DOI:** 10.1007/s00253-015-6729-4

**Published:** 2015-06-20

**Authors:** Charles H. D. Williamson, Luke A. Jain, Brajendra Mishra, David L. Olson, John R. Spear

**Affiliations:** Department of Civil and Environmental Engineering, Colorado School of Mines, Golden, CO 80401 USA; The Center for Microbial Genetics and Genomics, Northern Arizona University, Flagstaff, AZ 86011 USA; Department of Metallurgy and Materials Engineering, Colorado School of Mines, Golden, CO 80401 USA; Shell Technology Center Houston, 3333 Hwy 6 South, Houston, TX 77082 USA

**Keywords:** Microbial diversity, Fuel-grade ethanol, Microbiologically influenced corrosion, Pyrosequencing

## Abstract

Microbially influenced corrosion (MIC) is a costly problem that impacts hydrocarbon production and processing equipment, water distribution systems, ships, railcars, and other types of metallic infrastructure. In particular, MIC is known to cause considerable damage to hydrocarbon fuel infrastructure including production, transportation, and storage systems, often times with catastrophic environmental contamination results. As the production and use of alternative fuels such as fuel-grade ethanol (FGE) increase, it is important to consider MIC of engineered materials exposed to these “newer fuels” as they enter existing infrastructure. Reports of suspected MIC in systems handling FGE and water prompted an investigation of the microbial diversity associated with these environments. Small subunit ribosomal RNA gene pyrosequencing surveys indicate that acetic-acid-producing bacteria (*Acetobacter* spp. and *Gluconacetobacter* spp.) are prevalent in environments exposed to FGE and water. Other microbes previously implicated in corrosion, such as sulfate-reducing bacteria and methanogens, were also identified. In addition, acetic-acid-producing microbes and sulfate-reducing microbes were cultivated from sampled environments containing FGE and water. Results indicate that complex microbial communities form in these FGE environments and could cause significant MIC-related damage that may be difficult to control. How to better manage these microbial communities will be a defining aspect of improving mitigation of global infrastructure corrosion.

## Introduction

The detrimental effects of microbial contamination of fuel systems have been well described as microbial activity causes biofouling, fuel degradation, and microbially influenced corrosion (MIC) (Little and Lee 2007; Gaylarde et al. 1999; Rajasekar et al. 2012; Passman 2013 and references therein). MIC is the biologically mediated deterioration of a material (e.g., low carbon steel, stainless steel, copper) and can be attributed to a number of different mechanisms including microbial production of corrosive substances such as organic acids and sulfides as well as microbially-mediated oxidation and/or reduction of metals (Little and Lee 2007; Lewandowski and Beyenal 2009). It has been estimated that MIC is responsible for a large proportion of corrosion damage of metallic infrastructure and that microbial corrosion leads to billions of dollars of damage just in the USA each year (Little and Lee 2007; Passman 2013 and references therein). The development of effective strategies for the prevention and mitigation of MIC has proven difficult. While the corrosive impacts of microbes in oil and gas industry infrastructure have been well documented, MIC in ‘alternative’ fuel environments has been less extensively evaluated.

In the USA, fuel-grade ethanol (FGE) production has increased from approximately 850 million gallons in 1990 to approximately 13 billion gallons in 2013 due to efforts to use renewable transportation fuels (Renewable Fuels Association 2014). Ethanol is added to gasoline to offset fossil fuel consumption and to oxygenate the fuel to promote more complete combustion and lower emissions (US DOE 2012). Exposing engineered materials (e.g., various steel alloys and grades used in pipelines, tanks, valves, ships, and railcars) to new fuels can produce unanticipated environmental degradation of the material, which may result in equipment failures. For example, carbon steel was found to be susceptible to stress corrosion cracking (SCC) when exposed to fuel-grade ethanol (Kane et al. 2005; Lou et al. 2010; Lou et al. 2009; Sridhar et al. 2006), and exposure of steels to fuel-grade ethanol has resulted in failures of storage tanks, transfer piping, and other equipment (Kane et al. 2004). Pitting corrosion of carbon steel in ethanolic environments has also been reported (Lou and Singh 2010). Although, the potential for MIC in environments exposed to FGE has not been widely considered; pipes, tanks, and equipment in pump sumps exposed to FGE and water have been subject to corrosion issues of a suspected microbial nature (personal communication with industry personnel; Wilson et al. 2011; Pollock 2012). At high concentrations, ethanol is thought to be toxic to microbes due to negative impacts on cell membranes (Taylor et al. 2012). However, ethanol can also serve as a carbon source and/or electron donor for sulfate-reducing bacteria, acid-producing bacteria, and other microbes associated with MIC (Madigan et al. 2014). Microbial metabolism of ethanol also results in the formation of metabolites such as acetic acid, propionate, and hydrogen, which can be metabolized by other corrosion-inducing microbiota.

Microbiota typically live as biofilms on the steel (or other) surfaces of fuel transmission or storage infrastructure where water is present. Water, a limiting factor for microbial growth, is commonly found in pipelines, tanks, and other areas in fuel storage and transportation systems (Passman 2013; Passman 2003); thus, the potential for MIC in these systems should be evaluated. The objective of this research was to investigate the microbial diversity that may impact corrosion in environments exposed to fuel-grade ethanol and water. Because ethanol can serve as both a carbon source and/or electron donor for many microbes and because industry personnel have reported suspected MIC issues in some ethanolic environments, we hypothesized that environments exposed to ethanolic fuels and water host microbes capable of metabolizing ethanol and associated compounds to influence corrosion. To date, the microbial diversity of environments associated with ethanolic fuels and the potential for microbial corrosion in ethanolic environments has not been thoroughly investigated. Samples collected from fuel tanks that contained fuel-grade ethanol and accumulated water or ethanol fuel blends were analyzed via both culture-independent molecular techniques (16S rRNA gene pyrosequencing) and cultivation methods to elucidate the types of microbes present in these systems and to provide insight into how these microbes may impact corrosion processes.

## Materials and methods

### Sample collection

**Fig. 1:**
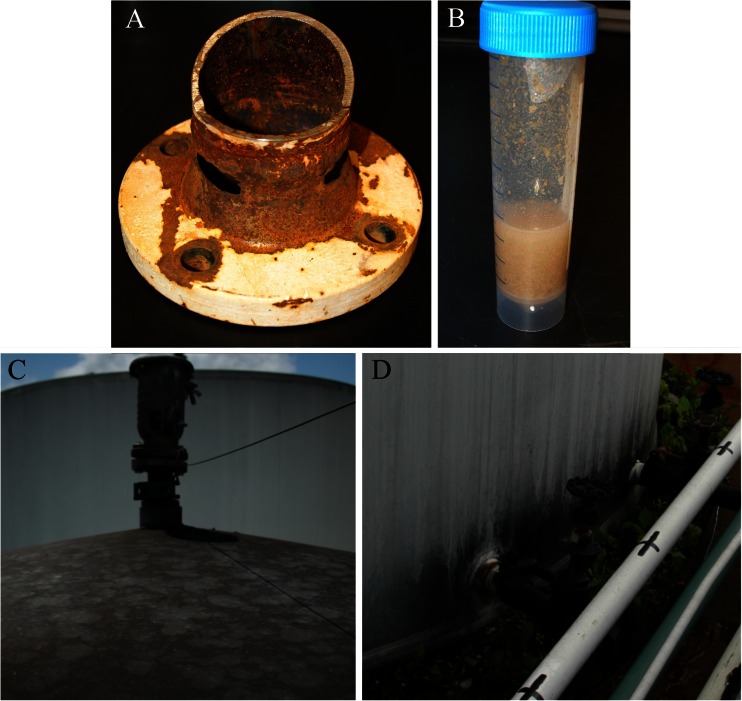
Images of equipment associated with FGE. **a** Corroded pipe flange removed from service after exposure to fuel-grade ethanol and water. **b** Bottoms/bulk liquid sampled from a tank containing fuel-grade ethanol and water (ECT.1). **c**, **d** Black biofilms formed on the outer surface of tanks containing E10 (EXT.1)

Samples were collected from six ethanol containment tanks over a time period of approximately 15 months (November 2009–February 2011). These ethanol containment tanks collect FGE as well as water (runoff, humidity, and/or condensate) during operations at fueling terminals. The sampled tanks contained significant amounts of water (estimated to be ~95 % water in some cases). Samples were obtained from the bottom of each tank. These samples included bulk liquid and solids collected aseptically into sterile, 50-mL tubes, stored on water ice at 4 °C. The pH of the liquid was measured with pH indicator strips. Additionally, one sample of suspected biofilm was collected from the exterior surface of an above ground storage tank that contained E10 fuel (a blend of 90 % gasoline and 10 % fuel-grade ethanol). A black film had formed on the painted exterior of the tank near safety valves and pipes exiting the tank (points on the tank where vapors likely escape). The formation of this black substance on tanks that contained FGE or ethanol fuel blends was reported to be a common occurrence by inspection personnel. The black substance was collected aseptically by scraping the substance into a 2-mL cryovial and frozen in liquid nitrogen in the field. Samples for DNA extraction were held at −80 °C until DNA was extracted. Samples for cultivation experiments were held at 4 °C until inoculation. Representative images of samples and sample locations are shown in Fig. 1. Ethanol containment tank samples are labeled ECT.1 through ECT.6; the sample from the exterior surface of the E10 fuel tank is labeled EXT.1.

### DNA extraction, PCR, and 454 pyrosequencing

DNA was extracted from ethanol containment tank samples (ECT.1–6) by a phenol:chloroform extraction similar to the procedure described by Dojka and colleagues (1998). Sample material was suspended in 500 μL Buffer A (200 mM Tris [pH 8.0], 50 mM EDTA, 200 mM NaCl), 200 μL 20 % sodium dodecyl sulfate, and 500 μL phenol-chloroform-isoamyl alcohol (24:24:1). Sterile garnet beads were added to the samples and were reciprocated on a Mini-Beadbeater (Biospec) at maximum speed for 1 min, extracted, and then extracted again with phenol-chloroform-isoamyl alcohol. DNA was precipitated with one volume isopropanol and 0.3 M sodium acetate. Precipitated DNA was washed with 70 % ethanol and suspended in nuclease-free water. DNA was extracted from sample EXT.1 using the MoBio Powersoil DNA extraction kit (MoBio, Carlsbad, CA); the 10-min vortexing step was replaced by 1 min of bead beating as described above. DNA was prepared for sequencing as described by Osburn and colleagues (2011) with the following exceptions: each qPCR reaction contained 4 μL of template DNA. qPCR reactions were performed in duplicate and pooled prior to normalization for sequencing. Primers (515F-modified 5′-GTGYCAGCMGCCGCGGTAA-3′ and 927R-modified 5′-CCGYCAATTCMTTTRAGTTT-3′; see Osburn et al. 2011) included adapter sequences for pyrosequencing on the GSFLX Titanium platform of the Roche 454 Pyrosequencing technology. Amplicons for each sample were normalized for sequencing with the SequalPrep Normalization Plate Kit (Invitrogen) (Harris et al. 2010), and pooled amplicons were gel purified with the EZNA Gel Extraction Kit (Omega BioTek). Sequencing was completed on the Roche 454 FLX Titanium platform at the EnGenCOre facility (now Selah Genomics) at the University of South Carolina.

Sequence analysis was carried out using the QIIME (Caporaso et al. 2010) and Mothur (Schloss et al. 2009) software packages. Initial quality filtering of the sequences included removing sequences with errors in barcodes or primers, homopolymer runs longer than 6 nucleotides, ambiguous base calls, or average quality scores less than 25. Sequences shorter than 400 nucleotides or longer than 500 nucleotides were also discarded. Initial quality control of sequencing data was based upon the findings of Huse et al. (2007). Sequences were denoised with DeNoiser version 1.4.0 (Reeder and Knight 2010). Chimeric sequences were identified by UCHIME (Edgar et al. 2011) and removed. Reverse primers were removed with a custom python script written by Charles Pepe-Ranney. Remaining sequences were aligned with SSU-ALIGN (Nawrocki 2009). Sequences failing to align to the appropriate alignment space were removed, and remaining sequences were trimmed to overlapping alignment coordinates using commands in Mothur (screen.seqs, filter.seqs, and summary.seqs—Schloss et al. 2009).

Sequences were clustered into operational taxonomic units (OTUs) with the average neighbor clustering method in Mothur (Schloss et al. 2009). Taxonomic classifications of sequences were assigned using the naïve Bayesian algorithm described by Wang and colleagues (2007) as implemented in Mothur (Schloss et al. 2009). The Silva SSURef108_NR database (Pruesse et al. 2007) was used as the training set for classifications. These reference sequences were quality filtered as described by Pepe-Ranney and colleagues (2012) and trimmed to the primer-targeted region of the 16S rRNA gene (515F-927R). Pyrosequences were also subjected to the Basic Local Alignment Search Tool (BLAST, Altschul et al. 1990) against the Silva SSURef108_NR database (Pruesse et al. 2007) and the National Center for Biotechnology Information (NCBI) non-redundant nucleotide database (Benson et al. 2005). Microbial community richness was estimated with Catchall (Bunge et al. 2012), and rarefaction curves were created in QIIME (Caporaso et al. 2010). Matplotlib (Hunter 2007) was used to create the heat map displayed in Fig. 3.

### Cultivation and identification of acetic-acid-producing and sulfate-reducing consortia

Two of the samples collected from ethanol containment tanks (ECT.1 and ECT.2) were used to inoculate media intended to grow acetic-acid-producing bacteria and sulfate-reducing bacteria. Acetic-acid-producing bacterial consortia were cultivated in an enrichment medium that contained glucose, 10 g/L; acetic acid, 3 mls/L; peptone, 15 g/L; yeast extract, 8 g/L; and ethanol, 5 % *v*/*v* (Lisdiyanti et al. 2003). After several culture transfers, the consortium was transferred to a medium that contained yeast extract, 0.5 g/L; peptone, 0.3 g/L; sodium chloride, 1 g/L; and ethanol, 5 % *v*/*v* (this medium was used for corrosion testing—see Jain 2011 and Sowards et al. 2014). All inoculated media were incubated with shaking at room temperature.

Sulfate-reducing bacterial consortia were cultivated from samples in a modified Postgate B medium (Postgate 1979) that contained potassium dihydrogen phosphate, 0.5 g/L; ammonium chloride, 1 g/L; calcium sulfate, 1 g/L; magnesium sulfate 7-hydrate, 2 g/L; yeast extract, 1 g/L; ascorbic acid, 0.1 g/L; thioglycolic acid, 0.1 g/L; and iron(II) sulfate 7-hydrate, 0.5 g/L. The pH of the medium was adjusted to 7.5 with sodium hydroxide. Filtered ethanol (2 % *v*/*v*) was added as a carbon source after autoclaving of the modified Postgate B medium. Cultures were incubated in serum vials with a nitrogen headspace at room temperature.

DNA was extracted from cultures with the phenol:chloroform extraction procedure described above. 16S rRNA gene amplification was carried out using primers 8F (5′-AGAGTTTGATCCTGGCTCAG-3′) and 1492R (5′-GGTTACCTTGTTACGACTT-3′) (Lane 1991). PCR, cloning, transformation, and sequencing were performed as described by Sahl and colleagues (2010). Sanger reads were called with PHRED (Ewing et al. 1998; Ewing and Green 1998) and assembled with PHRAP (www.phrap.org) via Xplorseq (Frank 2008). Identification of cultivar sequences was accomplished by parsimony addition into the Silva SSURef108_NR database (Pruesse et al. 2007) via ARB (Ludwig et al. 2004) and BLAST (Altschul et al. 1990) searches against the Silva SSURef108_NR database and the NCBI non-redundant nucleotide database (Benson et al. 2005). To more thoroughly establish phylogenetic relationships of cultivar and environmental sequences, maximum likelihood phylogenetic trees were created with RAxML (Stamatakis 2006). Closely related, relevant sequences were retrieved from Genbank, binned (97 % identity with UClust (Edgar et al. 2011) in QIIME (Caporaso et al. 2010)) and aligned and masked with SSU-ALIGN (Nawrocki 2009). Trees were created using the gamma distribution of rate heterogeneity and GTR substitution model, and the necessary number of bootstrap replicates (rapid bootstrapping method (Stamatakis et al. 2008)) was determined by using the RAxML frequency-based criterion (Pattengale et al. 2010). Redundant sequences and pyrosequences with close identity to cultivar sequences were aligned and masked as described above and added to the phylogenetic tree with pplacer (Matsen et al. 2010).

The acetic-acid-producing and sulfate-reducing consortia were also grown in the presence of A36 carbon steel coupons. Biofilm formation on the steel coupons was observed with scanning electron microscopy. After incubation, cells were fixed with 2 % glutaraldehyde/paraformaldehyde in phosphate buffered saline, dehydrated in an ethanol series, and chemically dried with hexamethyldisilazane. Samples were gold-sputtered and scanning electron microscopy was conducted on a FEI QUANTA 600I ESEM.

Cultivar sequences (Sanger sequencing data) have been submitted to Genbank under accession numbers KF661336-KF661341. Pyrosequencing data have been deposited in the NCBI Sequence Read Archive (BioProject ID PRJNA276558, study accession number SRP055553)

## Results

### Sample description

Samples collected from the bottoms of ethanol containment tanks (ECT samples) included bulk liquid and solids from tanks that contained fuel-grade ethanol and water. pH values of these bulk samples ranged from 4 to 7 (see Table 1). Operating personnel noted the smell of vinegar (acetic acid) upon sample collection. Corrosion damage was associated with 4 of the 6 ethanol containment tanks in the form of visible rust and rust-colored solutions (see Table 1). Sample EXT.1 included black solids scraped from painted, exterior tank surfaces near pressure-release valves.

**Table 1:** Sample information, pyrosequencing information, and biodiversity metrics

Sample ID/library name	Corrosion damage associated with tank	Dominant phylotype	pH	# of sequences	Observed OTUs	Estimated total species	Lower Cb^d^	Upper Cb^d^
ECT.1	Yes	*Acetobacter*	4	3526	32	32.1	22.6	57.3
ECT.2	Yes	*Acetobacter*	4.5	3235	104	132.3	96.2	208.7
ECT.3	No^a^	*Acetobacter*	4.5	1624	57	94.2	70.8	139.5
ECT.4	No^b^	*Sediminibacterium*	7	1197	139	585.9	297.4	1377.2
ECT.5	Yes	*Denitratisoma*	7	1077	77	177.5	126.6	280.9
ECT.6	Yes	*Acetobacter*	4.5	3309	30	25.5	20.7	38.5
EXT.1	No^c^	*Modestobacter*	NA	1375	18	19.5	18.3	24.7

^a^New tank

^b^Fiberglass tank

^c^Painted surface

^d^Catchall 95% confidence bound (Bunge et al. 2012)

### Pyrosequencing results

To identify microbes present in tank environments associated with fuel-grade ethanol, we generated small subunit ribosomal RNA (16S rRNA) gene libraries via pyrosequencing technology. Pyrosequence libraries included a total of approximately 15,000 partial 16S rRNA gene sequence reads with an average of approximately 2000 sequences per sample. Libraries are labeled according to the sample from which they were produced (ECT—ethanol containment tanks, EXT—tank exterior biofilm). Sample descriptions, pyrosequencing library names, and microbial diversity estimates are summarized in Table 1.

**Fig. 2:**
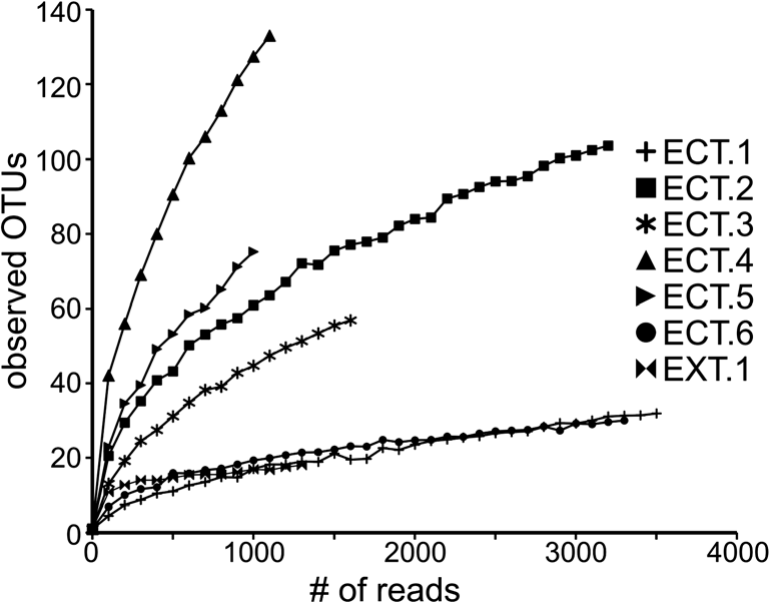
Rarefaction analysis for pyrosequencing libraries. Sequences were clustered with the average neighbor method in Mothur (Schloss et al. 2009) at a distance of 0.03. Rarefaction curves were created with QIIME (Caporaso et al. 2010)

Rarefaction curves (Fig. 2) indicate that samples ECT.4 and ECT.5 have the highest species (OTU) richness of the collected samples. Interestingly, these two samples had pH values of approximately seven while the other ethanol containment tank samples had pH values of 4–4.5 and lower species richness. This trend has also been observed in microbial communities from soils (Fierer and Jackson 2006; Lauber et al. 2009). Samples ECT.1, ECT.6, and EXT.1 exhibit lower species richness than other samples, and rarefaction analysis reveals that most of the diversity in these samples has been observed with the number of sequences generated for each sample. Parametric estimates of OTU richness for each sample were estimated with Catchall (Bunge et al. 2012) and are displayed in Table 1. These estimates also indicate that samples ECT.4 and ECT.5 display the highest OTU richness, while samples ECT.1, ECT.6, and EXT.1 have lower richness.

**Fig. 3:**
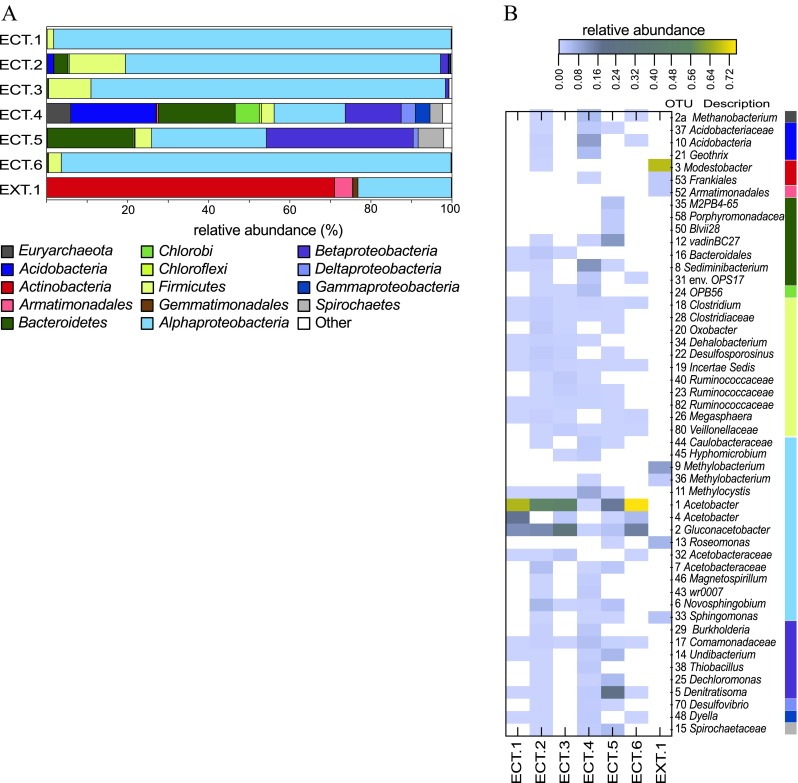
**a** Distribution of 16S rRNA gene pyrosequence reads for each sample library. Reads are distributed into phyla except for the *Proteobacteria*, which are distributed into classes. **b** Heat map displaying the 50 most abundant OTUs in the pyrosequencing libraries. The relative abundance of each OTU is indicated by the color of the *rectangles* in the figure—*yellow* rectangles indicate the most abundant OTU’s (see scale—*top right*). Taxonomic classifications of OTU’s are displayed to the *right* of the heat map. The *bar* on the *right side* of Fig. 3b corresponds to phyla/class designations in Fig. 3a

The majority of the pyrosequences in all 6 ethanol containment tank sample libraries represent members of the *Proteobacteria*, and four of the six ethanol containment tank samples (ECT.1, 2, 3, and 6) are dominated by members of the *Alphaproteobacteria* that include microbes closely related to *Acetobacter* spp. and *Gluconacetobacter* spp. (Fig. 3). These types of acetic-acid-producing microbes are also present in the ECT.4 and ECT.5 samples. The most abundant phylotype (OTU 1) in samples ECT.1, ECT.2, ECT.3, and ECT.6 has 100 % sequence identity to *Acetobacter lovaniensis* (accession number NR_114845, Cleenwerck et al. 2002). Abundant acetic-acid-producing bacterial sequences also include OTU 4, which has >99 % sequence identity to *Acetobacter aceti* (accession number NR_026121, Sievers et al. 1994), and OTU 2, which has >99 % sequence identity to *Gluconacetobacter liquefaciens* (accession number NR_026132, Sievers et al. 1994).

The pH of samples ECT.4 and ECT.5 was approximately 7, and neither of the most abundant phylotypes in these samples are putative acetic-acid-producing microbes. The most prevalent phylotype in the ECT.4 pyrosequencing library (OTU 8) is a member of the *Chitinophagaceae* family of the *Bacteroidetes* that has >99 % sequence identity to an uncultured bacterium identified in groundwater contaminated with oil (accession # KJ645492). The second most abundant phylotype (OTU 10) in ECT.4 is a member of the *Acidobacterium* with >99 % identity to an uncultured *Acidobacterium* identified in a microbial fuel cell (accession # AB286269, Ishii et al. 2008). The ECT.4 pyrosequencing library also includes sequences closely related to *Methanobacterales* spp. (OTU 2a), which are methanogenic members of the *Euryarchaeota* and have been found in environments including low-pH mining water treatment reactors (>99 % sequence identity to accession # EU717093). Sample ECT.4 also contains sequences (OTU 11) closely related to *Methylocystis* spp., which have been identified in soils at landfills (>99 % similar to accession # EU647259, Lin et al. 2009) and microbial fuel cell anodes (>99 % sequence identity to accession # JF326062). *Methylocystis* spp. are C1 metabolizers (i.e., consume methane) and include methanotrophic members of the *Alphaproteobacteria* (Bowman et al. 1993).

The most abundant phylotype in the ECT.5 pyrosequencing library (OTU 5) is a member of the *Betaproteobacteria* classified as a member of the *Denitratisoma*. This phylotype has >99 % identity to a bacterium isolated from wild rice (accession # AY235688). OTU 1 (100 % identity to *Acetobacter lovaniensis*) is the second most abundant phylotype in sample ECT.5. The third most abundant phylotype (OTU 12) in sample ECT.5 is a member of the *Bacteroidetes* (classified as a member of the *Rikenellaceae* family) that is closely related to microbes identified in iron-reducing consortia (accession # DQ676996, Lin et al. 2007), microbes that are found in urban storm water environments (accession # HE659028, Badin et al. 2012), and microbes that are present in environments contaminated with chlorinated hydrocarbons (accession # HM481376, Lee et al. 2012; accession # JF502581, Wei and Finneran 2011).

In addition to the microbes described above, the libraries from the ECT samples include numerous phylotypes putatively capable of a number of different metabolisms; microbes that utilize ethanol or metabolic products related to ethanol (e.g., acetate) are common. For instance, anaerobic members of the *Clostridiales* order (within the *Firmicutes* phylum) including *Clostridium* spp., *Desulfosporosinus* spp. and members of the *Ruminococcaceae* family, which have been identified in bioreactors designed to convert ethanol into n-caproic acid (Agler et al. 2012), are present in the ECT libraries. Members of the *Clostridiales* order include spore-forming microbes that are capable of surviving harsh conditions such as exposure to high concentrations of solvents (e.g., fuel-grade ethanol) (Vos et al. 2009).

The sequence library obtained from the biofilm sample from a painted exterior surface of a tank that contained E10 fuel (EXT.1) was dominated by members of the *Actinobacteria* and *Alphaproteobacteria* (Fig. 3). The most abundant phylotype (OTU 3, ~68 % of the library) is a member of the *Actinobacteria* classified as a *Modestobacter* spp. and shares 100 % sequence identity to an uncultured bacterium identified in samples of snow on which dust had been deposited due to storm events (accession #HQ396618, Chuvochina et al. 2011) and >99 % sequence identity to an uncultured bacterium identified in subsurface sediments (accession # HM186639, Lin et al. 2012). The *Alphaproteobacteria* in the pyrosequencing library from sample EXT.1 included *Methylobacterium* spp. (OTU 9—>99 % sequence identity to a *Methylobacterium* spp. identified in a concrete surface biofilm—accession # JN020186, Ragon et al. 2011) and a member of the *Roseomonas* (OTU 13) that has >99 % sequence identity to an uncultured bacterium identified in epilithic biofilms (accession # JQ627531, Ragon et al. 2012). *Alphaproteobacterial* members of sample EXT.1 also include sequences closely related to *Sphingomonas* spp. (OTU 33—>99 % identity to a groundwater biofilm clone, accession # FJ204468). Many microbes identified in sample EXT.1 are closely related to chemoorganoheterotrophs.

### Cultivation of acetic-acid-producing bacteria

**Fig. 4:**
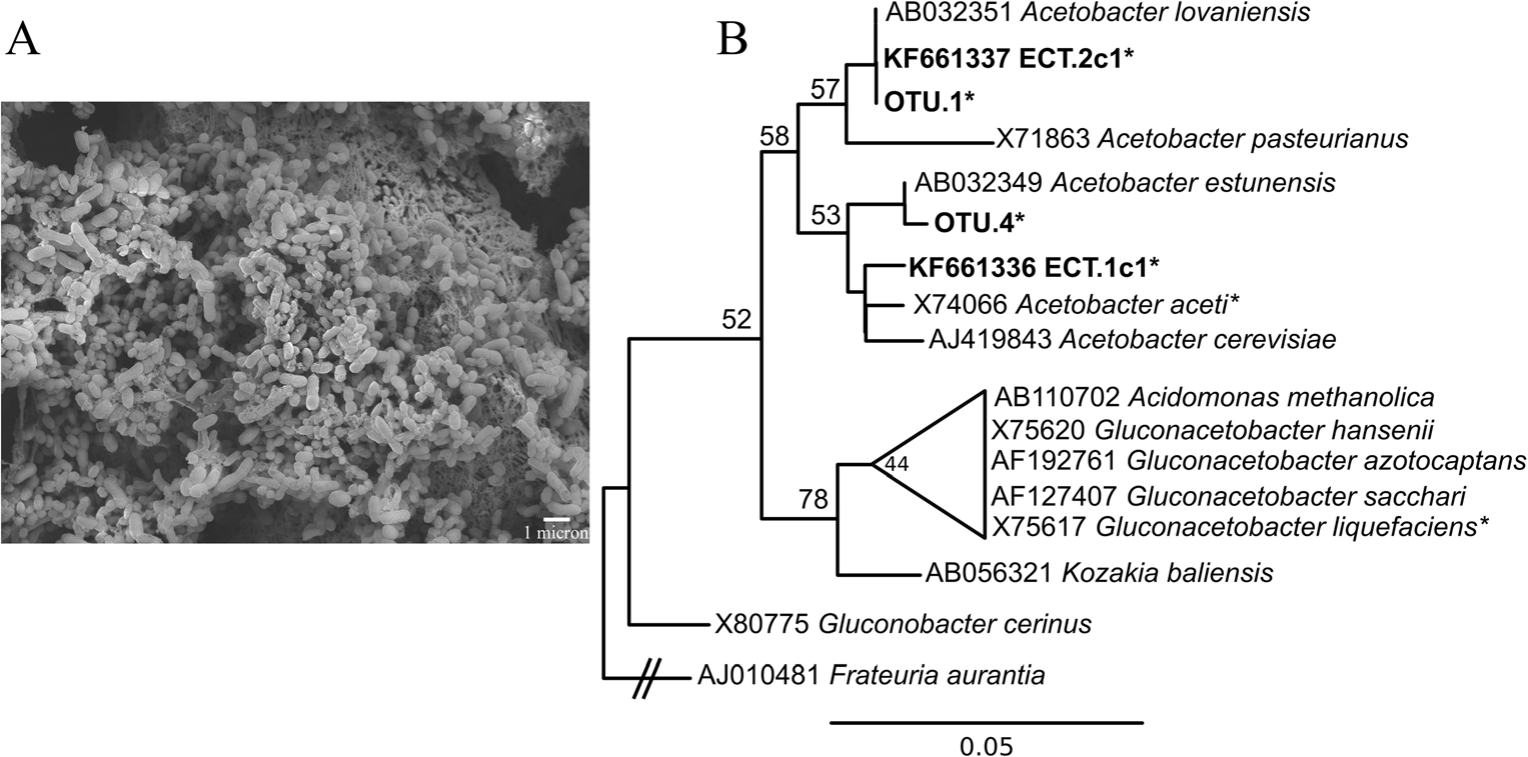
**a** Scanning electron micrograph of the acetic-acid-producing culture from sample ECT.1 on a steel coupon. **b** Maximum likelihood phylogenetic tree of members of the *Acetobacteraceae*. Sequences produced in this study are in *bold*. *Starred sequences* were inserted with pplacer (Matsen et al. 2010). Sequences representing members of the acetic-acid-producing cultures are labeled ECT.1c1 and ECT.2c1. OTUs from pyrosequencing libraries that are closely related to cultivar sequences are also included. Clades with bootstrap values below 50 % were collapsed. *Frateuria aurantia* (accession # AJ010481) was used as the outgroup (see Kersters et al. 2006)

Acetic-acid-producing bacteria were successfully cultivated from both ECT samples (ECT.1 and ECT.2) used for inoculation. Decreasing pH values were indicative of acetic acid production (though other organic acids may also have been produced). pH values of cultures decreased to approximately pH 3 (from approximately pH 6.5). Sanger sequenced small subunit (SSU) rRNA genes indicated that cultivars from both samples are closely related to *Acetobacter* spp. (Fig. 4). Both cultivar sequences are closely related to sequences in pyrosequencing libraries produced from environmental samples. The sequence from the ECT.1 sample acetic-acid-producing culture (sequence ECT.1c1) has >99 % identity to *Acetobacter aceti* (accession # NR_113549, Iino et al. 2012) and is closely related to OTU 4 (>99 % identity), which comprises ~18 % of the pyrosequencing library generated from sample ECT.1. The sequence from the ECT.2 sample acetic-acid-producing culture (sequence ECT.2c1) has 100 % BLAST identity to *Acetobacter lovaniensis* (accession # NR_114845, Cleenwerck et al. 2002), and is related to OTU 1 (100 % identity), which accounts for ~53 % of the pyrosequencing library from sample ECT.2.

### Cultivation of sulfate-reducing consortium

**Fig. 5:**
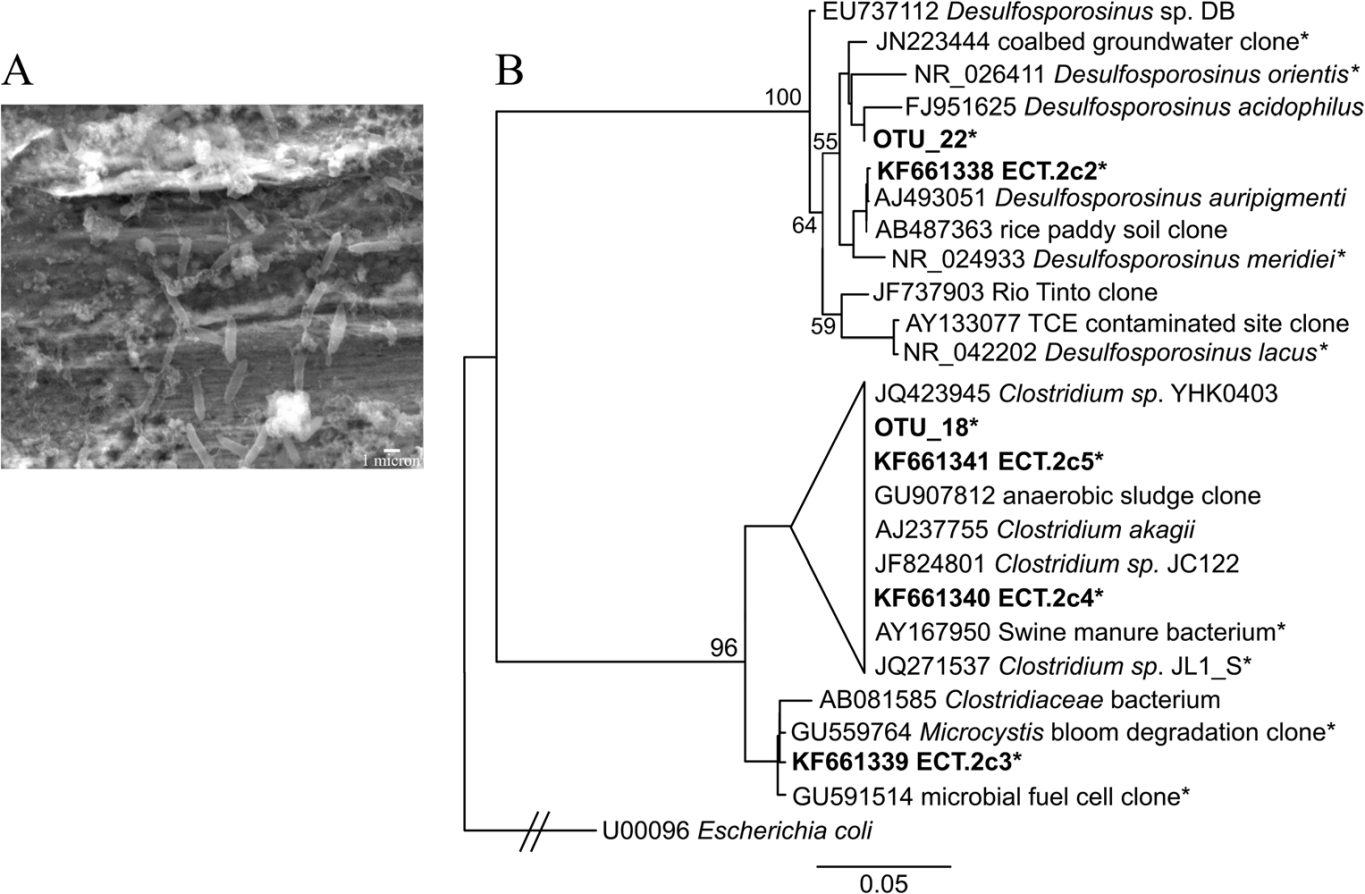
**a** Scanning electron micrograph of the sulfate-reducing consortium from sample ECT.2 on a steel coupon. **b** Maximum likelihood phylogenetic tree of members of the *Firmicutes*. Sequences produced in this study are in *bold*. *Starred sequences* were inserted with pplacer (Matsen et al. 2010). Sequences representing members of the sulfate-reducing consortium are labeled ECT.2c2-5. OTUs from pyrosequencing libraries that are closely related to cultivar sequences are also included. Clades with bootstrap values below 50 % were collapsed. *Escherichia coli* (accession # U00096) was used as the outgroup

Blackening of the modified Postgate B culture medium was indicative of growth of sulfate-reducing microbes, and sulfate reduction was observed only in vials inoculated with sample ECT.2. 16S rRNA gene Sanger sequence analyses indicated that the sulfate-reducing consortium included microbes closely related to *Desulfosporosinus* spp. as well as *Clostridium* spp., both of which are present in the ECT.2 pyrosequencing library (Fig. 5). Four phylotypes were identified in the sulfate-reducing consortium (sequences ECT.2c2, ECT.2c3, ECT.2c4, and ECT.2c5). Sequence ECT.2c2 is >99 % identical to *Desulfosporosinus auripigmenti* (accession # NR_025551 Stackebrandt et al. 2003) and is closely related (>97 % identity) to OTU 22, which comprises ~2 % of the ECT.2 pyrosequencing library. Sequences ECT.2c3, ECT.2c4, and ECT.2c5 group within the *Clostridiaceae* family. Sequences ECT.2c3 and ECT.2c4 are less than 97 % identical to any OTU present in the ECT.2 pyrosequencing library. Sequence ECT.2c3 is closely related (>99 % identity) to an uncultured bacterium clone identified from an anaerobic fermentation of a Microcystis bloom (accession # GU559764, Xing et al. 2011), and sequence ECT.2c4 is >99 % identical to a sulfate-reducing cultivar from the acidic Tinto River (accession # JQ271537, Sánchez-Andrea et al. 2012). Sequence ECT.2c5 is >99 % identical to a *Clostridium* sp. isolated from under a corroding gas pipeline (accession # JQ423945) and is closely related (>99 % identity) to OTU 18, which comprises ~1 % of the ECT.2 pyrosequencing library.

## Discussion

Reports of suspected MIC issues in environments in which carbon steel and other metal alloys are exposed to fuel-grade ethanol and water prompted the examination of the microbial diversity associated with these environments. As ethanol may serve as a carbon source and/or electron donor for microbial metabolisms, we hypothesized that these environments host microbial life capable of impacting corrosion processes through the production of organic acids, if not the oxidation/reduction of the metal alloys themselves. 16S rRNA gene pyrosequencing analyses indicate that microbes suspected to be capable of utilizing ethanol (and metabolites of ethanol) are present in all sampled environments. Additionally, cultivation experiments demonstrated that both acetic-acid-producing bacteria and sulfate-reducing bacteria inhabit these environments. Both culture-independent and culture-dependent methods identified microbes that have been associated with microbial corrosion (i.e., acid-producing and sulfate-reducing microbes) in ethanol containment tank samples.

Acetic-acid-producing microbes (*Acetobacter* spp. and *Gluconacetobacter* spp.) were the dominant members of most (4 of 6) of the ECT pyrosequencing libraries and were present in all of the ECT samples. Also, *Acetobacter* spp. were cultivated from both ECT samples (ECT.1 and ECT.2) used for inoculation. *Acetobacter* spp. and *Gluconacetobacter* spp. are strictly aerobic microbes capable of oxidizing ethanol to acetic acid and may also convert acetic acid to carbon dioxide and water (Kersters et al. 2006). The tank samples in which acetic-acid-producing microbes were the most prevalent phylotypes in the pyrosequencing libraries had pH values of approximately 4, indicating that the activities of these microbes had a considerable impact on the internal tank environment. It is important to note that the chemical environment of bulk phase tank contents may be different than the conditions at the fuel-water-tank interface where biofilm formation likely occurs. This interface (e.g., on the steel walls of a tank) is where the MIC process often initiates and persists. At the fuel-water-tank interface, all of the necessities of life are present: a carbon source, water, an electron donor (the hydrocarbon/ethanol blend and/or metals in the tank), and an electron acceptor such as O_2_ or previously oxidized metal (e.g., rusted steel). *Acetobacter* spp. are present in a variety of environments and have been reported to contaminate ethanolic environments such as fuel-grade ethanol fermentations (Heist 2009) and wines (Du Toit and Pretorius 2002; Bartowsky and Henschke 2008); thus, the presence of these microbes in fuel industry infrastructure exposed to ethanol and water is likely. Reports have indicated that acetic-acid-producing microbes may play a role in corrosion of pumping equipment at fueling stations (Wilson et al. 2011; Pollock 2012). An industry report associated microbial acetic acid production with corrosion of underground storage tanks containing ultra-low-sulfur diesel (Batelle Memorial Institute 2012).

Organic acids, including acetic acid, are byproducts of many microbial metabolisms. Microbial organic acid production has been shown to enhance corrosion or deterioration of many materials (Little and Lee 2007). Fungal and bacterial organic acid production has been associated with fuel degradation and corrosion of carbon steel, stainless steel, aluminum, and other materials that are used to construct fuel storage and transportation infrastructure (Salvarezza et al. 1983; Little et al. 1992; Little et al. 1995; McNamara et al. 2005). Acetic acid production by *Acetobacter aceti* has been shown to accelerate corrosion of cathodically protected stainless steel (Little et al. 1988), and corrosion of carbon steel has also been associated with microbial acetic acid production (Little et al. 1992). The corrosive nature of the *Acetobacter* spp. cultivated in this study is described in associated works elsewhere (Jain 2011; Sowards et al. 2014; Sowards and Mansfield 2014). Jain (2011) found that carbon steel exposed to an acetic-acid-producing bacterial culture experienced pitting corrosion, and Sowards and colleagues (2014) demonstrated that an *Acetobacter* spp. culture enhanced fatigue crack growth rates of carbon steels commonly used for pipelines and tanks. Sowards and Mansfield (2014) investigated the corrosion impact of biotically produced acetic acid on copper and steel in tests designed to simulate underground storage tank pump sumps. Corrosion damage was observed for both materials after direct exposure to the *Acetobacter* spp. culture as well as exposure to acetic acid vapor in the headspace above the cultures. Interestingly, acetic acid has been shown to impact the corrosion and cracking behavior of carbon steel in fuel-grade ethanol environments containing low amounts of water (abiotic tests) (Lou et al. 2009; Lou and Singh 2010). The research of Lou and colleagues did not address MIC; however, microbial acetic acid production could potentially impact these phenomena.

While fuels contain dissolved oxygen and oxic conditions are likely present in parts of a fuel tank environment (Passman 2003), anoxic micro-niches are likely to occur due to microbial oxygen consumption as has been demonstrated in biofilms (Costerton et al. 1995). The presence of anoxic micro-environments and anaerobic microbes suggests that interactions of many types of microbial metabolisms may impact corrosion in these FGE environments; thus, it is important to consider microbial communities when attempting to control MIC. Anaerobic microbes identified in the ECT samples include members of the *Proteobacteria* and *Firmicutes*. *Clostridium* spp. were identified in ECT sample pyrosequencing libraries as well as the ECT.2 sulfate-reducing consortium. Some *Clostridium* spp. are known to produce acetic acid from hydrogen and carbon dioxide (Braun et al. 1981), compounds likely to be present in the tanks due to the activity of *Acetobacter* spp. and *Gluconacetobacter* spp. Additionally, these aerobic, acetic-acid-producing microbes are likely to consume oxygen and create conditions suitable for the growth of anaerobic *Clostridium* spp. as well as sulfate-reducing bacteria such as *Desulfosporosinus* spp., which were also identified in pyrosequencing libraries as well as the sulfate-reducing consortium cultivated from sample ECT.2. The impact that the sulfate-reducing consortium cultivated in this study may have on corrosion of carbon steel are examined elsewhere (Jain 2011; Sowards et al. 2014), but briefly, Jain (2011) found that the sulfate-reducing consortium increased general corrosion rates of X52 and X70 linepipe steel. Sowards et al. (2014) demonstrated increased fatigue crack growth rates of A36, X52, and X70 steels in the presence of the sulfate-reducing consortium. *Clostridium* spp. are known to be associated with corrosion of carbon steels in oil and gas industry environments (Jan-Roblero et al. 2008; Monroy et al. 2011; Zhu et al. 2003). Microbial sulfate reduction has long been associated with MIC of many materials (Von Wolzogen Kuehr and van der Vlugt 1934; Little and Lee 2007; Javaherdashti 2008; Enning et al. 2012; Venzlaff et al. 2013), indicating that even if the corrosive impact of microbial acetic acid production was neutralized, microbial corrosion issues could persist due to microbial sulfate reduction or due to microbial utilization of iron and other alloy metals as a dominant electron donor. While sulfate-reducing microbes are often associated with oil industry environments (Cord-Ruwisch et al. 1987; Magot et al. 2000; Stevenson et al. 2011), we did find these types of microbes in these fuel-grade ethanol environments.

Methanogenic *Archaea* were identified in 4 of the 6 ECT sample pyrosequencing libraries. The most abundant methanogen identified (OTU 2a) is closely related to members of *Methanobacterium* including the cultured *Methanobacterium congolense* (Cuzin et al. 2001). Acidophilic methanogens have been described (Bräuer et al. 2006). Kotsyurbenko and colleagues (2007) described an acid-tolerant, hydrogenotrophic methanogen of the *Methanobacterium* genus isolated from acidic peat bogs. These types of methanogens as well as acetoclastic methanogens may thrive in ethanolic environments also containing acid-producing microbes. Methanogenic *Archaea* have been linked to elemental iron oxidation and corrosion (Dinh et al. 2004; Uchiyama et al. 2010). Zhang and colleagues (2003) suggested that hydrogenotrophic methanogens from a marine biofilm were directly responsible for mild steel corrosion while the acetoclastic methanogens were not directly responsible for corrosion but grew syntrophically with sulfate-reducing bacteria. Nelson and colleagues (2010) linked an increase in methanogens to the conversion of ethanol to acetate in soil column experiments designed to investigate the impact of ethanol-based fuels on microbial communities. Environments present in ethanolic fuel conveyance systems may provide niches in which methanogens thrive; however, the impact of methanogens on corrosion and deterioration of fuel industry infrastructure is not currently well understood. Methanogens may contribute to MIC as well as potentially play a role in substrate (e.g., hydrocarbons or ethanol) degradation in a tank or pipeline resulting in the production of volatile methane.

The pyrosequencing library created from the biofilm found on the external surface of the E10 fuel tank (sample EXT.1) is comprised of putatively chemoorganoheterotrophic phylotypes that have been associated with epilithic biofilms. The most prevalent OTU is closely related to *Modestobacter* spp., an *Actinobacterium* that has been identified on degrading stone surfaces (Eppard et al. 1996; Urzi 2004). Ragon and colleagues (2011) identified *Methylobacterium* spp., *Roseomonas* spp., and *Sphingomonas* spp., which were also present in the EXT pyrosequencing library, in biofilms sampled from concrete surfaces. Similar black crust biofilms may also be seen near exhaust vents of breweries. Such organisms could reside as endoliths within the pore spaces of concrete or as biofilms on the surface but in either case could contribute significantly to the weathering of the concrete. Gundlapally and Garcia-Pichel (2006) identified *Modestobacter* spp., *Methylobacterium* spp., *Roseomonas* spp., and *Sphingomonas* spp. in soil crusts. While some *Methylobacterium* spp. and *Sphingomonas* spp. have been shown to metabolize ethanol (Zhao et al. 2008; Šmejkalová et al. 2010), it is unclear if the biofilms forming on the external surfaces of these tanks utilize fuel escaping the tanks or if they are contributing to a MIC process from the outside of the tank, even though the tanks have a protective paint layer (we were not able to fully analyze the paint and underlying steel for evidence of corrosion). Phylotypes known to convert ethanol to acetic acid are not found in the EXT sample. While some *Methylobacterium* spp. and *Sphingomonas* spp. have been associated with copper corrosion (Pavissich et al. 2010), the actual corrosive nature of the biofilms sampled in this study was not investigated.

In summary, reports of suspected MIC of materials exposed to FGE and water prompted the investigation of microbial communities in these environments. Microbial communities associated with tanks that contain FGE and significant amounts of water included microbes capable of metabolizing ethanol and producing corrosive organic acids as well as microbes associated with other biocorrosion mechanisms (e.g., sulfate reduction). Though low water availability and high solvent content (fuel) may inhibit microbial activity under ideal operating conditions in many parts of fuel storage and transportation systems, microbial conversion of ethanol to acetic acid could potentially enhance corrosion of steels and other materials in systems (e.g., tanks, pipes, pump sumps) in which FGE and water are present. Putative acetic-acid producers *Acetobacter* spp. and *Gluconacetobacter* spp. are prevalent in pyrosequencing libraries derived from tank samples containing FGE and water. The presence of anaerobes such as sulfate-reducing bacteria suggests that syntrophs may impact corrosion in these environments. Future research is needed to more thoroughly understand microbial corrosion in many fuel environments.

## References

[CR1] Agler MT, Spirito CM, Usack JG, Werner JJ, Angenent LT (2012). Chain elongation with reactor microbiomes: upgrading dilute ethanol to medium-chain carboxylates. Energy Environ Sci.

[CR2] Altschul SF, Gish W, Miller W, Myers EW, Lipman DJ (1990). Basic local alignment search tool. J Mol Biol.

[CR3] Badin AL, Mustafa T, Bertrand C, Monier A, Delolme C, Geremia RA, Bedell JP (2012). Microbial communities of urban stormwater sediments: the phylogenetic structure of bacterial communities varies with porosity. FEMS Microbiol Ecol.

[CR4] Bartowsky EJ, Henschke PA (2008). Acetic acid bacteria spoilage of bottled red wine—a review. Int J Food Microbiol.

[CR5] Batelle Memorial Institute (2012). Corrosion in systems storing and dispensing ultra low sulfur diesel (ULSD), hypotheses investigation.

[CR6] Benson DA, Karsch-Mizrachi I, Lipman DJ, Ostell J, Wheeler DL (2005). GenBank. Nucleic Acids Res.

[CR7] Bowman JP, Sly LI, Nichols PD, Hayward AC (1993). Revised taxonomy of the methanotrophs: description of *Methylobacter* gen. nov., emendation of M*ethylococcus*, validation of M*ethylosinus* and *Methylocystis* species, and a proposal that the family *Methylococcaceae* includes only the group I methanotrophs. Int J Syst Bacteriol.

[CR8] Bräuer SL, Cadillo-Quiroz H, Yashiro E, Yavitt JB, Zinder SH (2006). Isolation of a novel acidiphilic methanogen from an acidic peat bog. Nature.

[CR9] Braun M, Mayer F, Gottschalk G (1981). *Clostridium aceticum* (Wieringa), a microorganism producing acetic acid from molecular hydrogen and carbon dioxide. Arch Microbiol.

[CR10] Bunge J, Woodard L, Böhning D, Foster JA, Connolly S, Allen HK (2012). Estimating population diversity with CatchAll. Bioinformatics.

[CR11] Caporaso JG, Kuczynski J, Stombaugh J, Bittinger K, Bushman FD, Costello EK, Fierer N, Peña AG, Goodrich JK, Gordon JI, Huttley GA, Kelley ST, Knights D, Koenig JE, Ley RE, Lozupone CA, McDonald D, Muegge BD, Pirrung M, Reeder J, Sevinsky JR, Turnbaugh PJ, Walters WA, Widmann J, Yatsunenko T, Zaneveld J, Knight R (2010). QIIME allows analysis of high-throughput community sequencing data. Nat Methods.

[CR12] Chuvochina MS, Alekhina IA, Normand P, Petit JR, Bulat SA (2011). Three events of Saharan dust deposition on the Mont Blanc glacier associated with different snow-colonizing bacterial phylotypes. Microbiology.

[CR13] Cleenwerck I, Vandemeulebroecke K, Janssens D, Swings J (2002). Re-examination of the genus *Acetobacter*, with descriptions of *Acetobacter cerevisiae* sp. nov. and *Acetobacter malorum* sp. nov. Int J Syst Evol Microbiol.

[CR14] Cord-Ruwisch R, Kleinitz W, Widdel F (1987). Sulfate-reducing bacteria and their activities in oil production. J Pet Technol.

[CR15] Costerton JW, Lewandowski Z, Caldwell DE, Korber DR, Lappin-Scott HM (1995). Microbial biofilms. Annu Rev Microbiol.

[CR16] Cuzin N, Ouattara AS, Labat M, Garcia JL (2001). *Methanobacterium congolense* sp. nov., from a methanogenic fermentation of cassava peel. Int J Syst Evol Microbiol.

[CR17] Dinh HT, Kuever J, Mußmann M, Hassel AW, Stratmann M, Widdel F (2004). Iron corrosion by novel anaerobic microorganisms. Nature.

[CR18] Dojka MA, Hugenholtz P, Haack SK, Pace NR (1998). Microbial diversity in a hydrocarbon- and chlorinated-solvent-contaminated aquifer undergoing intrinsic bioremediation. Appl Environ Microbiol.

[CR19] Du Toit WJ, Pretorius IS (2002). The occurrence, control and esoteric effect of acetic acid bacteria in winemaking. Ann Microbiol.

[CR20] Edgar RC, Haas BJ, Clemente JC, Quince C, Knight R (2011). UCHIME improves sensitivity and speed of chimera detection. Bioinformatics.

[CR21] Enning D, Venzlaff H, Garrelfs J, Dinh HT, Meyer V, Mayrhofer K, Hassel AW, Stratmann M, Widdel F (2012). Marine sulfate-reducing bacteria cause serious corrosion of iron under electroconductive biogenic mineral crust. Environ Microbiol.

[CR22] Eppard M, Krumbein WE, Koch C, Rhiel E, Staley JT, Stackebrandt E (1996). Morphological, physiological, and molecular characterization of *Actinomycetes* isolated from dry soil, rocks, and monument surfaces. Arch Microbiol.

[CR23] Ewing B, Green P (1998). Base-calling of automated sequencer traces using phred. II. Error probabilities. Genome Res.

[CR24] Ewing B, Hillier L, Wendl MC, Green P (1998). Base-calling of automated sequencer traces using phred. I. Accuracy assessment. Genome Res.

[CR25] Fierer N, Jackson RB (2006). The diversity and biogeography of soil bacterial communities. Proc Natl Acad Sci U S A.

[CR26] Frank DN (2008). XplorSeq: a software environment for integrated management and phylogenetic analysis of metagenomic sequence data. BMC Bioinf.

[CR27] Gaylarde CC, Bento FM, Kelley J (1999). Microbial contamination of stored hydrocarbon fuels and its control. Rev Microbiol.

[CR28] Gundlapally S, Garcia-Pichel F (2006). The community and phylogenetic diversity of biological soil crusts in the Colorado Plateau studied by molecular fingerprinting and intensive cultivation. Microb Ecol.

[CR29] Harris JK, Sahl JW, Castoe TA, Wagner BD, Pollock DD, Spear JR (2010). Comparison of normalization methods for construction of large, multiplex amplicon pools for next-generation sequencing. Appl Environ Microbiol.

[CR30] Heist P (2009) Identifying, controlling the most common microbial contaminants. Ethanol Producer Magazine March 5, 2009. EthanolProducer.com.

[CR31] Hunter JD (2007). Matplotlib: a 2D graphics environment. Comput Sci Eng.

[CR32] Huse S, Huber J, Morrison H, Sogin M, Welch MD (2007). Accuracy and quality of massively parallel DNA pyrosequencing. Genome Biol.

[CR33] Iino T, Suzuki R, Kosako Y, Ohkuma M, Komagata K, Uchimura T (2012). *Acetobacter okinawensis* sp. nov., *Acetobacter papayae* sp. nov., and *Acetobacter persicus* sp. nov.; novel acetic acid bacteria isolated from stems of sugarcane, fruits, and a flower in Japan. J Gen Appl Microbiol.

[CR34] Ishii S, Shimoyama T, Hotta Y, Watanabe K (2008). Characterization of a filamentous biofilm community established in a cellulose-fed microbial fuel cell. BMC Microbiol.

[CR35] Jain LA (2011). Evaluation of the propensity for microbiologically influenced corrosion of steels in fuel grade ethanol environments.

[CR36] Jan-Roblero J, Posadas A, de la Serna JZD, García R, Hernández-Rodríguez C (2008). Phylogenetic characterization of bacterial consortia obtained of corroding gas pipelines in Mexico. World J Microbiol Biotechnol.

[CR37] Javaherdashti R (2008). Microbiologically influenced corrosion: an engineering insight.

[CR38] Kane RD, Maldonado JG, Klein LJ (2004) Stress corrosion cracking in fuel ethanol: a newly recognized phenomenon. NACE International.

[CR39] Kane RD, Sridhar N, Brongers MP, Bea JA, Agrawal AK, Klein LJ (2005). Stress corrosion cracking in fuel ethanol: a recently recognized phenomenon. Mater Perform.

[CR40] Kersters K, Lisdiyanti P, Komagata K, Swings J, Dworkin M, Falkow S, Rosenberg E, Schleifer KH, Stackebrandt E (2006). The family *Acetobacteraceae*: the genera *Acetobacter*, *Acidomonas*, *Asaia*, *Gluconacetobacter*, *Gluconobacter*, and *Kozakia*. The Prokaryotes.

[CR41] Kotsyurbenko OR, Friedrich MW, Simankova MV, Nozhevnikova AN, Golyshin PN, Timmis KN, Conrad R (2007). Shift from acetoclastic to H2-dependent methanogenesis in a west Siberian peat bog at low pH values and isolation of an acidophilic *Methanobacterium* strain. Appl Environ Microbiol.

[CR42] Lane DJ, Stackebrandt E, Goodfellow M (1991). 16S/23S rRNA sequencing. Nucleic acid techniques in bacterial systematics.

[CR43] Lauber CL, Hamady M, Knight R, Fierer N (2009). Pyrosequencing-based assessment of soil pH as a predictor of soil bacterial community structure at the continental scale. Appl Environ Microbiol.

[CR44] Lee PKH, Warnecke F, Brodie EL, Macbeth TW, Conrad ME, Andersen GL, Alvarez-Cohen L (2012). Phylogenetic microarray analysis of a microbial community performing reductive dechlorination at a TCE-contaminated site. Environ Sci Technol.

[CR45] Lewandowski Z, Beyenal H, Flemming HC, Murthy PS, Venkatesan R, Cooksey K (2009). Mechanisms of microbially influenced corrosion. Mar. Ind. Biofouling.

[CR46] Lin B, Hyacinthe C, Bonneville S, Braster M, Van Cappellen P, Röling WFM (2007). Phylogenetic and physiological diversity of dissimilatory ferric iron reducers in sediments of the polluted Scheldt estuary, Northwest Europe. Environ Microbiol.

[CR47] Lin B, Monreal CM, Tambong JT, Miguez CB, Carrasco-Medina L (2009). Phylogenetic analysis of methanotrophic communities in cover soils of a landfill in Ontario. Can J Microbiol.

[CR48] Lin X, Kennedy D, Fredrickson J, Bjornstad B, Konopka A (2012). Vertical stratification of subsurface microbial community composition across geological formations at the Hanford Site. Environ Microbiol.

[CR49] Lisdiyanti P, Katsura K, Potacharoen W, Navarro R, Yamada Y, Uchimura T, Komagata K (2003). Diversity of acetic acid bacteria in Indonesia, Thailand, and the Philippines. Microbiol Cult Collect.

[CR50] Little BJ, Lee JS (2007). Microbiologically influenced corrosion.

[CR51] Little B, Wagner P, Duquette D (1988). Technical note: microbiologically induced increase in corrosion current density of stainless steel under cathodic protection. Corrosion.

[CR52] Little B, Wagner P, Mansfeld F (1992). An overview of microbiologically influenced corrosion. Electrochim Acta.

[CR53] Little B, Ray R, Hart K, Wagner P (1995). Fungal-induced corrosion of wire rope. Mater Perform.

[CR54] Lou X, Singh PM (2010). Role of water, acetic acid and chloride on corrosion and pitting behaviour of carbon steel in fuel-grade ethanol. Corros Sci.

[CR55] Lou X, Yang D, Singh PM (2009). Effect of ethanol chemistry on stress corrosion cracking of carbon steel in fuel-grade ethanol. Corrosion.

[CR56] Lou X, Yang D, Singh PM (2010). Film breakdown and anodic dissolution during stress corrosion cracking of carbon steel in bioethanol. J Electrochem Soc.

[CR57] Ludwig W, Strunk O, Westram R, Richter L, Meier H, Yadhukumar Buchner A, Lai T, Steppi S, Jobb G, Förster W, Brettske I, Gerber S, Ginhart AW, Gross O, Grumann S, Hermann S, Jost R, König A, Liss T, Lüßmann R, May M, Nonhoff B, Reichel B, Strehlow R, Stamatakis A, Stuckmann N, Vilbig A, Lenke M, Ludwig T, Bode A, Schleifer KH (2004). ARB: a software environment for sequence data. Nucleic Acids Res.

[CR58] Madigan MT, Martinko JM, Bender KS, Buckley DH, Stahl DA (2014). Brock biology of microorganisms.

[CR59] Magot M, Ollivier B, Patel BKC (2000). Microbiology of petroleum reservoirs. Antonie Van Leeuwenhoek.

[CR60] Matsen FA, Kodner RB, Armbrust EV (2010). pplacer: linear time maximum-likelihood and Bayesian phylogenetic placement of sequences onto a fixed reference tree. BMC Bioinf.

[CR61] McNamara CJ, Perry TD, Leard R, Bearce K, Dante J, Mitchell R (2005). Corrosion of aluminum alloy 2024 by microorganisms isolated from aircraft fuel tanks. Biofouling.

[CR62] Monroy OAR, Gayosso MJH, Ordaz NR, Olivares GZ, Ramírez CJ (2011). Corrosion of API XL 52 steel in presence of *Clostridium celerecrescens*. Mater Corros.

[CR63] Nawrocki EP (2009). Structural RNA homology search and alignment using covariance models.

[CR64] Nelson DK, LaPara TM, Novak PJ (2010). Effects of ethanol-based fuel contamination: microbial community changes, production of regulated compounds, and methane generation. Environ Sci Technol.

[CR65] Osburn MR, Sessions AL, Pepe-Ranney C, Spear JR (2011). Hydrogen-isotopic variability in fatty acids from Yellowstone National Park hot spring microbial communities. Geochim Cosmochim Acta.

[CR66] Passman FJ (2003). Fuel and fuel system microbiology—fundamentals, diagnosis, and contamination control.

[CR67] Passman FJ (2013). Microbial contamination and its control in fuels and fuel systems since 1980—a review. Int Biodeterior Biodegrad.

[CR68] Pattengale ND, Alipour M, Bininda-Emonds ORP, Moret BME, Stamatakis A (2010). How many bootstrap replicates are necessary?. J Comput Biol.

[CR69] Pavissich JP, Vargas IT, González B, Pastén PA, Pizarro GE (2010). Culture dependent and independent analyses of bacterial communities involved in copper plumbing corrosion. J Appl Microbiol.

[CR70] Pepe-Ranney C, Berelson WM, Corsetti FA, Treants M, Spear JR (2012). *Cyanobacterial* construction of hot spring siliceous stromatolites in Yellowstone National Park. Environ Microbiol.

[CR71] Pollock S (2012) Accelerated corrosion of UST equipment (an ethanol hangover). Presented at the 23rd National Tank Conference, St. Louis, Missouri, March 19–21, 2011

[CR72] Postgate JR (1979). The sulphate-reducing bacteria.

[CR73] Pruesse E, Quast C, Knittel K, Fuchs BM, Ludwig W, Peplies J, Glöckner FO (2007). SILVA: a comprehensive online resource for quality checked and aligned ribosomal RNA sequence data compatible with ARB. Nucleic Acids Res.

[CR74] Ragon M, Restoux G, Moreira D, Møller AP, López-García P (2011). Sunlight-exposed biofilm microbial communities are naturally resistant to Chernobyl ionizing-radiation levels. PLoS One.

[CR75] Ragon M, Fontaine MC, Moreira D, López-García P (2012). Different biogeographic patterns of prokaryotes and microbial eukaryotes in epilithic biofilms. Mol Ecol.

[CR76] Rajasekar A, Maruthamuthu S, Ting YP, Balasubramanian R, Rahman PKSM, Singh SN (2012). Bacterial degradation of petroleum hydrocarbons. Microb. Degrad. Xenobiotics.

[CR77] Reeder J, Knight R (2010). Rapid denoising of pyrosequencing amplicon data: exploiting the rank-abundance distribution. Nat Methods.

[CR78] Renewable Fuels Association (2014) Renewable Fuels Association Statistics. http://www.ethanolrfa.org/pages/statistics. Accessed 4 Nov 2014

[CR79] Sahl JW, Fairfield N, Harris JK, Wettergreen D, Stone WC, Spear JR (2010). Novel microbial diversity retrieved by autonomous robotic exploration of the world’s deepest vertical phreatic sinkhole. Astrobiology.

[CR80] Salvarezza RC, de Mele MFL, Videla HA (1983). Mechanisms of the microbial corrosion of aluminum alloys. Corrosion.

[CR81] Sánchez-Andrea I, Rojas-Ojeda P, Amils R, Sanz JL (2012). Screening of anaerobic activities in sediments of an acidic environment: Tinto River. Extremophiles.

[CR82] Schloss PD, Westcott SL, Ryabin T, Hall JR, Hartmann M, Hollister EB, Lesniewski RA, Oakley BB, Parks DH, Robinson CJ, Sahl JW, Stres B, Thallinger GG, Horn DJV, Weber CF (2009). Introducing mothur: open-source, platform-independent, community-supported software for describing and comparing microbial communities. Appl Environ Microbiol.

[CR83] Sievers M, Ludwig W, Teuber M (1994). Phylogenetic positioning of *Acetobacter*, *Gluconobacter*, *Rhodopila* and *Acidiphilium* species as a branch of acidophilic bacteria in the α-subclass of *Proteobacteria* based on 16S ribosomal DNA sequences. Syst Appl Microbiol.

[CR84] Šmejkalová H, Erb TJ, Fuchs G (2010). Methanol assimilation in *Methylobacterium extorquens* AM1: demonstration of all enzymes and their regulation. PLoS One.

[CR85] Sowards JW, Mansfield E (2014). Corrosion of copper and steel alloys in a simulated underground storage-tank sump environment containing acid-producing bacteria. Corros Sci.

[CR86] Sowards JW, Williamson CHD, Weeks TS, McColskey JD, Spear JR (2014). The effect of *Acetobacter* sp. and a sulfate-reducing bacterial consortium from ethanol fuel environments on fatigue crack propagation in pipeline and storage tank steels. Corros Sci.

[CR87] Sridhar N, Price K, Buckingham J, Dante J (2006). Stress corrosion cracking of carbon steel in ethanol. Corrosion.

[CR88] Stackebrandt E, Schumann P, Schüler E, Hippe H (2003). Reclassification of *Desulfotomaculum auripigmentum* as *Desulfosporosinus auripigmenti* corrig., comb. nov. Int J Syst Evol Microbiol.

[CR89] Stamatakis A (2006). RAxML-VI-HPC: maximum likelihood-based phylogenetic analyses with thousands of taxa and mixed models. Bioinformatics.

[CR90] Stamatakis A, Hoover P, Rougemont J (2008). A rapid bootstrap algorithm for the RAxML web servers. Syst Biol.

[CR91] Stevenson BS, Drilling HS, Lawson PA, Duncan KE, Parisi VA, Suflita JM (2011). Microbial communities in bulk fluids and biofilms of an oil facility have similar composition but different structure. Environ Microbiol.

[CR92] Taylor M, Ramond JB, Tuffin M, Burton S, Eley K, Cowan D, Liu ZL (2012). Mechanisms and applications of microbial solvent tolerance. Microb. Stress Toler. Biofuels..

[CR93] Uchiyama T, Ito K, Mori K, Tsurumaru H, Harayama S (2010). Iron-corroding methanogen isolated from a crude-oil storage tank. Appl Environ Microbiol.

[CR94] Urzi C (2004). Microbial deterioration of rocks and marble monuments of the Mediterranean Basin: a review. Corros Rev.

[CR95] US DOE (2012) Alternative fuels data center: ethanol fuel basics. http://www.afdc.energy.gov/fuels/ethanol_fuel_basics.html. Accessed 9 Nov 2012

[CR96] Venzlaff H, Enning D, Srinivasan J, Mayrhofer KJJ, Hassel AW, Widdel F, Stratmann M (2013). Accelerated cathodic reaction in microbial corrosion of iron due to direct electron uptake by sulfate-reducing bacteria. Corros Sci.

[CR97] Von Wolzogen Kuehr CAH, van der Vlugt LS (1934). Graphitization of cast iron as an electrobiochemical process in anaerobic soils. Water.

[CR98] Vos P, Garrity G, Jones D, Krieg NR, Ludwig W, Rainey FA, Schleifer KH, Whitman WB (2009). Bergey’s manual of systematic bacteriology: Volume 3: The *Firmicutes*.

[CR99] Wang Q, Garrity GM, Tiedje JM, Cole JR (2007). Naïve Bayesian classifier for rapid assignment of rRNA sequences into the new bacterial taxonomy. Appl Environ Microbiol.

[CR100] Wei N, Finneran KT (2011). Influence of ferric iron on complete dechlorination of trichloroethylene (TCE) to ethene: Fe(III) reduction does not always inhibit complete dechlorination. Environ Sci Technol.

[CR101] Wilson JT, Adair CJ, Paul C, Wilkin RT, Skender JP, Azadpour-Keeley A, Liang Z, Barbery A, Fowler E, Strauss R, West K, Hickey J, and Hansen R. Relationship between ethanol in fuel and corrosion in STP sumps. Presented at 2011 EPA region 3 LUST technical workshop, Rehoboth Beach, DE, September 28–30, 2011

[CR102] Xing P, Guo L, Tian W, Wu QL (2011). Novel *Clostridium* populations involved in the anaerobic degradation of *Microcystis* blooms. ISME J.

[CR103] Zhang T, Fang HHP, Ko BCB (2003). Methanogen population in a marine biofilm corrosive to mild steel. Appl Microbiol Biotechnol.

[CR104] Zhao HP, Wang L, Ren JR, Li Z, Li M, Gao H-W (2008). Isolation and characterization of phenanthrene-degrading strains *Sphingomonas* sp. ZP1 and *Tistrella* sp. ZP5. J Hazard Mater.

[CR105] Zhu XY, Lubeck J, Kilbane JJ (2003). Characterization of microbial communities in gas industry pipelines. Appl Environ Microbiol.

